# A Two-Year Longitudinal Study of the Association between Oral Frailty and Deteriorating Nutritional Status among Community-Dwelling Older Adults

**DOI:** 10.3390/ijerph18010213

**Published:** 2020-12-30

**Authors:** Masanori Iwasaki, Keiko Motokawa, Yutaka Watanabe, Maki Shirobe, Hiroki Inagaki, Ayako Edahiro, Yuki Ohara, Hirohiko Hirano, Shoji Shinkai, Shuichi Awata

**Affiliations:** 1 Tokyo Metropolitan Institute of Gerontology, Tokyo 173-0015, Japan; kemotokawa@gmail.com (K.M.); ywata@den.hokudai.ac.jp (Y.W.); mshirobe@tmig.or.jp (M.S.); inagaki@tmig.or.jp (H.I.); aedahiro@tmig.or.jp (A.E.); yohara@tmig.or.jp (Y.O.); h-hiro@gd5.so-net.ne.jp (H.H.); sshinkai@tmig.or.jp (S.S.); awata@tmig.or.jp (S.A.); 2 Gerodontology, Department of Oral Health Science, Faculty of Dental Medicine, Hokkaido University, Sapporo 060-8586, Japan; 3 School of Nutritional Sciences, Kagawa Nutrition University, Saitama 350-0288, Japan

**Keywords:** epidemiology, geriatrics, longitudinal study, nutritional status, oral health

## Abstract

Background: Limited longitudinal studies exist to evaluate whether poor oral health and functions affect the incidence of deteriorating nutritional status. We investigated if there were longitudinal associations between oral frailty, defined as accumulated deficits in oral health, and deteriorating nutritional status among community-dwelling older adults. Methods: The study population consisted of 191 men and 275 women (mean age, 76.4 years) from the Takashimadaira Study. Multifaced oral health assessment was performed at baseline, and oral frailty was defined as having ≥3 of the following six components: fewer teeth, low masticatory performance, low articulatory oral motor skill, low tongue pressure, and difficulties in chewing and swallowing. Nutritional status assessment was performed at baseline and two-year follow-up using the Mini Nutritional Assessment^®^-Short Form (MNA^®^-SF). Deteriorating nutritional status was defined as a decline in the nutritional status categories based on the MNA^®^-SF score during the study period. The association between oral frailty and deteriorating nutritional status was assessed using logistic regression analyses. Results: Oral frailty was observed in 67 (14.4%) participants at baseline. During the study, 58 (12.4%) participants exhibited deteriorating nutritional status. After adjusting for potential confounders, oral frailty was significantly associated with deteriorating nutritional status (adjusted odds ratio, 2.24; 95% confidence interval, 1.08–4.63). Conclusion: Community-dwelling older adults with oral frailty had an increased risk of deteriorating nutritional status.

## 1. Introduction

To date, studies have demonstrated that poor oral health and functions are associated with malnutrition among older adults [1,2,3,4,5]. Potential explanations for this relationship include the effect of insufficient dietary intake caused by masticatory and swallowing disorders [6,7]. However, previous studies investigated only a single aspect of oral health. Because individual oral health problems are interrelated and their prevalence increases with aging, older adults frequently have coexisting oral health problems [8,9]. Recently, oral frailty, which is defined operationally as accumulated deficits in oral health, has been introduced in Japan [10]. We have already reported the cross-sectional association between oral frailty and malnutrition among community-dwelling older adults [9].

Although it was a novel finding, the major shortcoming of this study was its cross-sectional design. Moreover, most previous studies investigating the association between oral health and functions with nutritional status had cross-sectional designs, which prevents us from clarifying the temporal sequence. Malnutrition is common in older adults and it increases the risks of frailty, sarcopenia, morbidity, and mortality [11,12,13,14,15,16]. Investigating the relationship between malnutrition and oral frailty in longitudinal, instead of cross-sectional, studies is important to gain insight into oral frailty as a potential risk factor for malnutrition. Such insights, in turn, may lead to new strategies from a dental perspective to prevent deteriorating nutritional status.

We followed a cohort of community-dwelling older adults for 2 years to investigate if there were longitudinal associations between oral frailty and deteriorating nutritional status in this population. Specifically, we hypothesized that older adults with oral frailty were more likely to suffer deteriorating nutritional status.

## 2. Materials and Methods

### 2.1. Study Population

This longitudinal study, with a two-year observation period (2016 to 2018), was a subset study of the Takashimadaira Study [17], which is an ongoing cohort study with the aims of developing a model for dementia-friendly communities in a metropolitan area. The study was initiated in 2016 and includes individuals aged ≥ 70 years listed in the basic resident register of Takashimadaira, Itabashi ward, Tokyo, Japan. Baseline examinations included assessments of nutritional and oral health status, a questionnaire survey, medical interview, and an anthropometric evaluation. In 2018, follow-up investigations to assess the nutritional status of participants were conducted. Individuals who were undernourished at the baseline did not participate in the follow-up investigation, and those who had missing data were excluded. Our study was conducted in full accordance with the ethical principles of the Declaration of Helsinki and was approved by the Ethics Committee of the Tokyo Metropolitan Institute of Gerontology (Approval Numbers: 9 and 31 in 2016). All participants provided written informed consent before participating.

### 2.2. Assessment of Nutritional Status

Nutritional status was assessed at baseline (2016) and follow-up (2018) using the Mini Nutritional Assessment^®^-Short Form (MNA^®^-SF) [18,19], which is comprised of six items: decreased food intake, involuntary weight loss, mobility, acute disease or psychological stress, neuropsychological problems, and body mass index (BMI). The MNA^®^-SF score ranges from 0 to 14. Study participants were categorized into three groups: normal nutritional status (score 12–14), at risk of malnutrition (score 8–11), and malnourished (score 0–7).

The study population consisted of those assessed as having normal nutritional status according to the baseline MNA^®^-SF score. Deteriorating nutritional status was defined if they became at risk of malnutrition or malnourished according to the MNA^®^-SF score at the two-year follow-up.

### 2.3. Assessment of Oral Health Status

Oral health status assessment was conducted by a trained dentist. The number of teeth and denture use were determined. Masticatory performance was assessed using β-carotene–containing gummy jelly (UHA; Mikakuto Co., Ltd., Osaka, Japan). Participants were asked to chew a gummy jelly for 30 strokes. Dentures, when used, were left on for assessment purpose. Using a fully automatic measuring device [20], the increase in the surface area of the ground gummy jelly in a square millimeter was measured and was used as a parameter of masticatory performance. Articulatory oral motor skill was evaluated by repetitive articulatory rate (oral diadochokinesis (oral-DDK)). The number of repetitions of the monosyllable ‘‘ta’’ per second was recorded using an oral function measuring device (Kenkou-Kun Handy; Takei Scientific Instruments Co., Ltd., Niigata, Japan) [21]. Tongue pressure (TP), which is the force produced by contact between the anterior part of the hard palate and tongue, was measured using the JMS Tongue Pressure Device (JMS Co., Ltd., Hiroshima, Japan) [22]. The mean value of three-time measurements of TP was used for analyses. Self-perceived difficulties in chewing and swallowing were assessed by the questions, “Do you have any difficulties chewing tough foods compared to six months ago?” and “Have you choked on your tea or soup recently?” on the Kihon Checklist developed by the Japanese Ministry of Health, Labor, and Welfare [23].

Participants were defined as having oral frailty if they had ≥3 of the following six components [9,10]: fewer teeth, low masticatory performance, low articulatory oral motor skill, low TP, and self-perceived difficulties in chewing and swallowing. The detailed criteria for each component are shown in Table 1.

### 2.4. Questionnaire Survey

Data on participants’ age, sex, educational status (i.e., years of schooling), income, smoking status, alcohol consumption, physical activity level, appetite, relationships with family and friends, living situation, instrumental activities of daily living (IADL), and depressive symptoms were obtained using a self-administered questionnaire. Subsequently, they were defined and categorized as follows: income was dichotomized as annual income of <3 million Japanese Yen or not (27,312 USD at the 2016 yearly-average exchange rate), smoking status as current smoker or not, and alcohol consumption as daily drinker or not. Physical activity levels were defined as low if participants answered “no” to the question, “Do you engage in physical exercise or sports?” Appetite was assessed using the Simplified Nutritional Appetite Questionnaire (SNAQ), with poor appetite defined as an SNAQ score ≤ 14 [24]. Social isolation was defined if participants answered “no” to the question, “Do you have contact at least once a week with anyone, including relatives living apart, friends, and neighbors?” [25]. IADL was assessed using the Japan Science and Technology Agency Index of Competence (JST-IC), with higher scores indicating greater competence [26]. Depressive symptoms were assessed using the Japanese version of the 15-item Geriatric Depression Scale (GDS-15) and were defined as a GDS-15 score of ≥6 [27].

### 2.5. Medical Interviews

Well-trained study staff confirmed the participants’ comorbidity status, the use of medication, and their cognitive status during the medical interviews. Nine comorbidities were identified, including heart disease, stroke, hypertension, respiratory diseases, digestive disorders, bone and joint diseases, Parkinson’s disease, diabetes, and cancer [9]. The number of medications was confirmed, and polypharmacy was defined as the concurrent use of ≥5 medications [28]. Cognitive status was assessed using the Mini-Mental State Examination (MMSE). Cognitive impairment was defined as a MMSE score of ≤23.

### 2.6. Anthropometric Measurement

Anthropometric measures included weight (kg) and height (cm). Individual BMI was calculated by dividing the weight in kilograms by the squared height in meters.

### 2.7. Statistical Analyses

Analyses were performed with the statistical software package STATA version 16.1 (StataCorp, College Station, TX, USA). The level of significance (2-tailed test) was set to 0.05.

Descriptive statistics were performed to characterize the study population and compare groups with and without oral frailty. In addition, baseline characteristics were compared between individuals who did and did not (lost to follow-up) participate in the follow-up investigations. The *t*-test, Mann–Whitney *U*-test, or χ^2^ test were used when appropriate.

Oral frailty was hypothesized to be associated with deteriorating nutritional status, and this hypothesis was assessed with univariable and multivariable logistic regression models. Models included deteriorating nutritional status as the outcome. In line with previous studies [1,29,30,31,32], the following baseline variables were considered covariates: MNA^®^-SF score, age, sex, educational status, income, smoking habit, drinking habit, physical activity level, appetite, social isolation, living situation, JST-IC score, number of comorbidities, polypharmacy, depressive symptoms, and cognitive impairment.

Three models were constructed. Model 1 included only oral frailty as an exposure (univariable model). Model 2 added variables associated significantly with deteriorating nutritional status in the univariable analysis. Model 3 included all covariates (full model). In addition, for sensitivity analyses, separate logistic regression analyses were performed to investigate the association between the number of oral frailty components and deteriorating nutritional status. The association between individual components of oral frailty and deteriorating nutritional status was also assessed using logistic regression analyses.

For all models, inverse probability weighting (IPW) was used to adjust for selection bias due to loss to follow-up [33]. Weights were calculated using baseline characteristics that showed significant differences (*p* < 0.05) between individuals who participated in the follow-up investigations and those lost to follow-up.

## 3. Results

**Figure 1:**
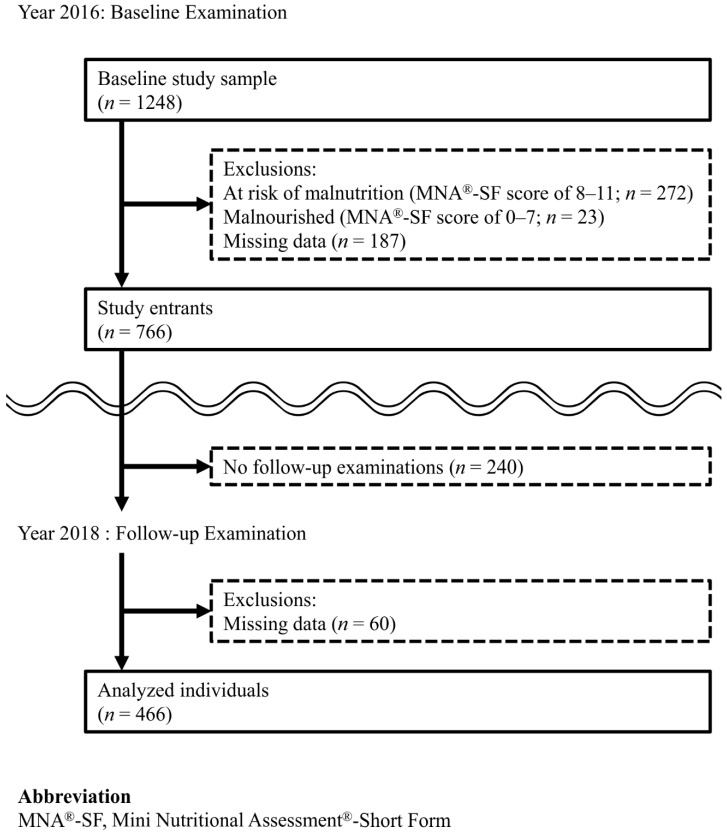
Flow diagram of our study.

The study population comprised 466 men and women. The selection process results were as follows and are shown in Figure 1. In 2016, 1248 individuals participated in the baseline examination. Of these individuals, 482 were excluded because they were at risk of malnutrition (*n* = 272), were malnourished (*n* = 23), or had missing data (*n* = 187), leaving 766 study entrants. In 2018, 240 study entrants did not participate in follow-up examinations. Table S1 shows the comparison of baseline variables between the individuals who did (*n* = 526) and did not (*n* = 240) participate in follow-up examinations. Variables that showed statistically significant differences between groups were number of teeth, masticatory performance, oral-DDK “ta”, TP, self-perceived difficulty in chewing, denture use, age, educational status, physical activity level, appetite, JST-IC, polypharmacy, depressive symptoms, and cognitive function. These variables were used for IPW calculation. Of the 526 individuals who participated in follow-up examinations, 60 had missing data and were excluded. Therefore, 466 individuals (191 men and 275 women; mean age (standard deviation [SD]), 76.4 (4.1) years) were included in the analyses.

Among the 466 individuals analyzed, 67 (14.4%) had oral frailty at baseline. Median (interquartile range (IQR)) number of oral frailty components was 1 (0–2). The prevalence of each oral frailty component ranged from 14.8% (low articulatory oral motor skill) to 32.2% (few remaining teeth; Table 1).

Table 2 shows the baseline characteristics according to the presence of oral frailty. Participants with oral frailty had fewer teeth and poorer oral function and were more likely to use dentures. In addition, those with oral frailty had a higher frequency of current smoking, poor appetite, and depressive symptoms and a lower JST-IC score, and they were less physically active.

Two-year follow-up nutritional assessments revealed that 408 of the 466 study participants maintained their nutritional status. On the other hand, 58 (12.4%) exhibited deteriorating nutritional status (56 became at risk of malnutrition and two became malnourished based on MNA^®^-SF score at follow-up).

Table 3 shows the association between different baseline health characteristics and deteriorating nutritional status. A higher MNA^®^-SF score and JST-IC score at baseline were significantly associated with lower odds of deteriorating nutritional status. In contrast, poor appetite and depressive symptoms were significantly associated with higher odds of deteriorating nutritional status.

Table 4 shows the results from the logistic regression models for the association between oral frailty and deteriorating nutritional status. The first column, Model 1, shows the regression model result with only oral frailty as a predictor. Oral frailty was associated significantly with deteriorating nutritional status (odds ratio (OR), 2.57; 95% confidence interval (CI), 1.32–4.99). The second column, Model 2, shows model results adding MNA^®^-SF score, poor appetite, JST-IC score, and depressive symptoms as covariates. Oral frailty remained statistically significant. Baseline MNA^®^-SF score and JST-IC score were also associated with deteriorating nutritional status. The third column shows the results of Model 3, modeling all variables (full model). The multivariate-adjusted OR (95% CI) for oral frailty was 2.24 (1.08–4.63). In addition, the effects of MNA^®^-SF score and JST-IC score remained significant.

Further logistic regression analyses showed the significant association between number of oral frailty components and deteriorating nutritional status. In Model 3 of Table S2, the multivariate-adjusted OR for the increase in one component of oral frailty was 1.30 (95% CI, 1.01–1.68). The association between individual component of oral frailty and deteriorating nutritional status is presented in Table S3. Although all components of oral frailty tended toward increased ORs for deteriorating nutritional status, none reached statistical significance in the fully adjusted model (Model 3).

## 4. Discussion

Community-dwelling older adults with oral frailty at baseline, defined as accumulated deficits in oral health, were more likely to exhibit deteriorating nutritional status at two-year follow-up. The significant association between oral frailty and deteriorating nutritional status remained significant after adjusting for other important baseline health characteristics. Furthermore, the accumulation of oral health deficits had a dose–response relationship with deteriorating nutritional status. In contrast, individual components of oral frailty did not have significant effects on deteriorating nutritional status. These findings indicated that it was the accumulation of multiple components of oral frailty rather than individual components that contributed to deteriorating nutritional status. To our knowledge, this is the first longitudinal study demonstrating that older adults with multiple oral health problems had increased risk of deteriorating nutritional status. Our results highlight the importance of conducting a comprehensive assessment of oral health status among older adults. Although further studies are needed to verify these conclusions, oral assessments meanwhile can be effective tools to detect older individuals at higher risk of deteriorating nutritional status in the community setting.

Malnutrition is associated with increased risks of frailty, sarcopenia, morbidity, and mortality [11,12,13,14,15,16]. Meanwhile, poor oral health reportedly was associated with frailty, sarcopenia, morbidity, and mortality [10,34]. Our results can be used as a basis for further studies to investigate whether malnutrition is an intermediate on the pathway between oral and systemic health.

Older adults with masticatory and swallowing dysfunction were likely to avoid foods that are difficult to chew, such as fruits, vegetables, and meat, which generally contain rich nutrients [7,35,36]. Previous studies have demonstrated that older adults with poor oral health had less intake of proteins, vitamins, and fiber [6,37,38]. In addition, poor oral health has been associated with poor diet quality [39] and a less varied diet [40]. Poor quantity and quality of diet can contribute to malnutrition [41]. Overall, a plausible longitudinal association was detected between oral frailty and malnutrition. Furthermore, the observed association agrees with that from previous studies that investigated individual oral condition and malnutrition [2,5].

At baseline, 14.4% of the study population had oral frailty. The epidemiologic study involving 2011 older Japanese reported a 15.9% prevalence of oral frailty [10]. There was no great discrepancy between our value and theirs.

We applied the operational definition of oral frailty introduced by the previous large-scale epidemiological study conducted by Tanaka et al. [10]. In this study, 16 oral status measurements were made with regard to the onset of adverse health outcomes such as physical frailty, sarcopenia, and disability. Eventually, six measures showed predictive ability for adverse health outcomes and were selected as components of oral frailty. Multicollinearity problems were not observed among these six components [10].

Besides oral frailty, poor IADL is associated with deteriorating nutritional status. Nutritional status and functional capacity are interrelated [42]. Reduced functional capacity predicted a decline in the nutritional status [43], whereas poor nutritional status predicted a decline in the functional capacity [44]. Our findings are in agreement with those from previous studies. In addition, poor appetite and depressive symptoms at the baseline were found to be associated with deteriorating nutritional status. These conditions can lead to inadequate dietary intake among older adults. Previous studies [30,31] have reported that poor appetite and depressive symptoms are associated with poor nutritional status. Our findings are in agreement with those from previous studies.

One distinction of this study with regard to earlier research is that we used rich data on oral health and functions. These data enabled us to define oral frailty based on a comprehensive measurement of oral health. We found a dose–response relationship between components of oral frailty and deteriorating nutritional status. Oral frailty has the potential to be a global measure of oral health. Another distinguishing factor is that we used a longitudinal design, thereby allowing us to determine the temporal association between oral frailty and malnutrition.

On the other hand, several limitations of our study must be considered. First, oral health and functions were affected by dental treatment. However, we could not obtain the information on dental treatment during follow-up. Therefore, we were unable to assess this effect. Second, no data on the development of diseases and the related treatment during the study period was obtained; therefore, we could not consider these effects in the statistical model. Third, the study population consisted of Japanese adults who participated in the survey voluntarily. The association between oral frailty and nutritional status should be tested in other groups to validate the generalizability of our results. Fourth, although we controlled for various potential confounders, residual confounding due to unmeasured variables, such as meal preparation support and practical cooking ability, and/or unexpected confounding variables may exist, as with any multivariable analysis. Finally, out of 766 study entrants, 240 individuals did not participate in the follow-up examination. Comparison of the study participants revealed that those who were lost to follow-up had poor oral health functions and general health. Although we applied IPW to adjust for the selection bias due to the loss to follow-up [33], it may not have been fully adjusted.

## 5. Conclusions

Our study demonstrated that older adults with oral frailty had increased risk of deteriorating nutritional status. Maintaining comprehensive oral health and function may be effective for the prevention of malnutrition in community-dwelling older adults. Future studies that explore effective approaches to control or reverse oral frailty and that assess whether such approaches have beneficial effects on nutritional status in older adults would be an interesting next step.

## Figures and Tables

**Table 1:** Six components of oral frailty used in this study.

Component	Prevalence, *n* (%)
1. Few remaining teeth: Number of teeth < 20	150 (32.2%)
2. Low masticatory performance: Increased surface area of comminuted gummy jelly; men, <1178 mm^2^; women, <1743 mm^2^)	78 (16.7%)
3. Low articulatory oral motor skill: Oral-DDK “ta”; men < 5.2 times/s; women, 5.4 times/s	69 (14.8%)
4. Low TP: men, <27.4 kPa; women, <26.5 kPa	81 (17.4%)
5. Difficulties chewing tough foods: An answer of “yes” to the question “Do you have any difficulties chewing tough foods compared to six months ago?”	70 (15.0%)
6. Difficulties in swallowing tea or soup: An answer of “yes” to the question “Have you choked on your tea or soup recently?”	108 (23.2%)

Oral-DDK, oral diadochokinesis; TP, tongue pressure.

**Table 2:** Baseline characteristics of the study population according to the presence of oral frailty.

	Total	Oral Frailty		
		(-)	(+)	
	*n* = 466	*n* = 399	*n* = 67	*p*-Value
*n* of teeth, median (IQR)	24 (16–27)	25 (20–27)	11 (6–18)	<0.01
Masticatory performance (mm^2^), median (IQR)	4396 (2570–5656)	4643 (3251–5834)	1343 (499–3374)	<0.01
Oral-DDK ‘‘ta’’ (times/s), median (IQR)	6.1 (0.8)	6.1 (0.7)	5.7 (1.1)	<0.01
TP (kPa), median (IQR)	33.7 (8.2)	34.3 (7.8)	30.4 (9.7)	<0.01
Difficulties eating tough foods, *n* (%)	70 (15.0%)	33 (8.3%)	37 (55.2%)	<0.01
Difficulties in swallowing tea or soup, *n* (%)	108 (23.2%)	74 (18.5%)	34 (50.7%)	<0.01
Denture use, *n* (%)	217 (46.6%)	163 (40.9%)	54 (80.6%)	<0.01
Age, mean (SD)	76.4 (4.1)	76.3 (4.1)	76.8 (4.3)	0.39
Sex, *n* (%)				0.35
Women	275 (59.0%)	232 (58.1%)	43 (64.2%)	
Men	191 (41.0%)	167 (41.9%)	24 (35.8%)	
Body weight (kg), mean (SD)	58.2 (8.9)	58.3 (9.1)	57.6 (8.3)	0.55
Height (cm), mean (SD)	156.2 (8.6)	156.4 (8.7)	154.9 (7.8)	0.21
BMI (kg/m^2^), mean (SD)	23.8 (2.6)	23.8 (2.6)	24.0 (2.7)	0.59
Educational status (years of schooling), median (IQR)	12 (12–16)	12 (12–16)	12 (12–14)	0.22
Annual income < 3 million JPY, *n* (%)	271 (58.2%)	225 (56.4%)	46 (68.7%)	0.06
Current smoker, *n* (%)	35 (7.5%)	26 (6.5%)	9 (13.4%)	0.05
Daily drinker, *n* (%)	62 (13.3%)	53 (13.3%)	9 (13.4%)	0.97
Low physical activity, *n* (%)	310 (66.5%)	258 (64.7%)	52 (77.6%)	0.04
Poor appetite, *n* (%)	165 (35.4%)	131 (32.8%)	34 (50.7%)	0.01
Social isolation, *n* (%)	178 (38.2%)	149 (37.3%)	29 (43.3%)	0.35
Living alone, *n* (%)	164 (35.2%)	134 (33.6%)	30 (44.8%)	0.08
JST-IC, median (IQR)	11 (9–13)	12 (10–13)	10 (9–12)	<0.01
Number of comorbidities, median (IQR)	2 (1–3)	2 (1–2)	2 (1–3)	0.10
Polypharmacy, *n* (%)	119 (25.5%)	104 (26.1%)	15 (22.4%)	0.52
Depressive symptoms, *n* (%)	60 (12.9%)	40 (10.0%)	20 (29.9%)	<0.01
Cognitive impairment, *n* (%)	19 (4.1%)	18 (4.5%)	1 (1.5%)	0.25

BMI, body mass index; IQR, interquartile range; JPY, Japanese Yen; JST-IC, Japan Science and Technology Agency Index of Competence; MNA^®^-SF, Mini Nutritional Assessment^®^-Short Form; oral-DDK, oral diadochokinesis; SD, standard deviation; TP, tongue pressure.

**Table 3:** Unadjusted ORs with 95% CIs for malnutrition in relation to study participants’ characteristics ^a^.

Variables ^b^	Unadjusted ORs	95% CIs	*p*-Value
MNA^®^-SF (per one increase)	0.40	(0.28–0.57)	<0.01
Age (per one increase)	1.02	(0.95–1.09)	0.58
Men (vs. women)	0.86	(0.48–1.54)	0.61
Years of schooling (per one increase)	0.94	(0.85–1.05)	0.29
Annual income < 3 million JPY	1.07	(0.60–1.91)	0.82
Current smoker	0.80	(0.23–2.79)	0.73
Daily drinker	1.40	(0.65–2.98)	0.39
Low physical activity level	1.51	(0.81–2.80)	0.20
Poor appetite	2.28	(1.29–4.04)	<0.01
Social isolation	1.33	(0.75–2.37)	0.33
Living alone	0.92	(0.51–1.69)	0.80
JST-IC score (per one increase)	0.85	(0.77–0.94)	<0.01
Number of comorbidities (per one increase)	1.10	(0.87–1.37)	0.42
Polypharmacy	1.16	(0.61–2.23)	0.65
Depressive symptoms	2.38	(1.19–4.76)	0.01
Cognitive impairment	2.41	(0.82–7.05)	0.11

CI, confidence interval; JST-IC, the Japan Science and Technology Agency Index of Competence; MNA^®^-SF, Mini Nutritional Assessment^®^-Short Form; OR, odds ratio. ^a^ Applying inverse probability weighting. ^b^ Except for age, sex, years of schooling, number of comorbidities, and JST-IC, ORs and CIs of being positive are presented.

**Table 4:** Three logistic regression models for the association between oral frailty and deteriorating nutritional status based on the MNA^®^-SF score ^a^.

	Outcome = Having MNA^®^-SF Score of < 12 at Two-Year Follow-Up Assessment								
	Model 1 (Oral Frailty Only)			Model 2 (Model 1 + Other Variables that Yielded *p*-Values < 0.05 in the Crude Model)			Model 3 (Fully Adjusted Model)		
Variables ^b^	ORs	95% CI	*p*-Value	ORs	95% CI	*p*-Value	ORs	95% CI	*p*-Value
Oral frailty	2.57	(1.32–4.99)	0.01	2.13	(1.05–4.33)	0.04	2.24	(1.08–4.63)	0.03
MNA^®^-SF (per one increase)				0.42	(0.28–0.63)	<0.01	0.43	(0.28–0.65)	<0.01
Age (per one increase)							1.01	(0.94–1.08)	0.86
Men (vs. women)							0.71	(0.36–1.39)	0.32
Years of schooling (per one increase)							1.03	(0.91–1.16)	0.64
Annual income < 3 million JPY							1.00	(0.47–2.14)	1.00
Current smoker							0.50	(0.16–1.57)	0.23
Daily drinker							1.47	(0.65–3.35)	0.36
Low physical activity level							1.26	(0.63–2.50)	0.52
Poor appetite				1.51	(0.82–2.79)	0.18	1.56	(0.85–2.85)	0.15
Social isolation							1.30	(0.68–2.47)	0.43
Living alone							0.61	(0.27–1.39)	0.24
JST-IC score (per one increase)				0.88	(0.79–0.98)	0.02	0.88	(0.78–0.99)	0.04
Number of comorbidities (per one increase)							1.05	(0.81–1.36)	0.70
Polypharmacy							1.07	(0.50–2.26)	0.87
Depressive symptoms				0.96	(0.42–2.19)	0.91	1.18	(0.49–2.86)	0.71
Cognitive impairment							2.03	(0.58–7.19)	0.27

CI, confidence interval; JPY, Japanese Yen; JST-IC, the Japan Science and Technology Agency Index of Competence; MNA^®^-SF, Mini Nutritional Assessment^®^-Short Form; OR, odds ratio. ^a^ Applying inverse probability weighting. ^b^ Except for age, sex, years of schooling, number of comorbidities, and JST-IC, ORs and CIs of being positive are presented.

## Data Availability

The data presented in this study are available on request from the corresponding author. The data are not publicly available due to ethicolegal restrictions imposed by the Ethics Committee at Tokyo Metropolitan Institute of Gerontology.

## Supplementary Materials

The following are available online at https://www.mdpi.com/1660-4601/18/1/213/s1, Table S1: Baseline characteristics of the study entrants with and without follow-up examinations, Table S2: Three logistic regression models for the association between the number of components of oral frailty and deteriorating nutritional status based on the MNA^®^-SF score.
