# Supplementary material for: A Two-Year Longitudinal Study of the Association between Oral Frailty and Deteriorating Nutritional Status among Community-Dwelling Older Adults
Source: Int J Environ Res Public Health. 2020 Dec 30;18(1):213. doi: 10.3390/ijerph18010213 (PMC7796237; doi:10.3390/ijerph18010213)
Supplement: Supplementary file 1 [file ijerph-18-00213-s001.pdf]

Table S1. Baseline characteristics of the study entrants with and without follow-up examinations

	Study entrants	Follow-up examinations		
		Did not participate (Dropout)	Participated	
	n = 766	n = 240	n = 526	p-value
Oral health status				
n of teeth, median (IQR)	23 (14–27)	21 (9–26)	24 (16–27)	<0.01
Masticatory performance (mm^2), median (IQR)	4129 (2121–5578)	3862 (1386–5167)	4396 (2556–5656)	<0.01
Oral-DDK "ta" (times/s), median (IQR)	6.0 (0.9)	5.9 (1.0)	6.1 (0.8)	0.04
TP (kPa), median (IQR)	33.2 (8.4)	32.2 (8.6)	33.6 (8.3)	0.02
Difficulties in eating tough foods, n (%)	129 (16.8%)	51 (21.3%)	78 (14.8%)	0.03
Difficulties in swallowing tea or soup, n (%)	167 (21.8%)	47 (19.6%)	120 (22.8%)	0.32
Denture use, n (%)	383 (50.0%)	136 (56.7%)	247 (47.0%)	0.01
Other characteristics				
Age, mean (SD)	76.8 (4.6)	77.6 (5.3)	76.4 (4.1)	<0.01
Sex, n (%)				0.10
Women	439 (57.3%)	127 (52.9%)	312 (59.3%)	
Men	327 (42.7%)	113 (47.1%)	214 (40.7%)	
MNA®-SF score	13 (12–14)	13 (12–14)	13 (13–14)	0.14
Body weight (kg), mean (SD)	58.1 (9.2)	58.2 (9.9)	58.1 (8.9)	0.95
Height (cm), mean (SD)	156.0 (8.5)	155.7 (8.3)	156.1 (8.5)	0.50
BMI (kg/m^2), mean (SD)	23.8 (2.8)	23.9 (3.2)	23.8 (2.6)	0.51

Educational Status (years of schooling), median (IQR)	12 (12–15)	12 (11–14)	12 (12–16)	0.01
Annual income < 3 million JPY, *n* (%)	459 (59.9%)	154 (64.2%)	305 (58.0%)	0.11
Current smoker, *n* (%)	54 (7.0%)	14 (5.8%)	40 (7.6%)	0.37
Daily drinker, *n* (%)	113 (14.8%)	40 (16.7%)	73 (13.9%)	0.31
Low physical activity, *n* (%)	535 (69.8%)	184 (76.7%)	351 (66.7%)	0.01
Poor appetite, *n* (%)	294 (38.4%)	107 (44.6%)	187 (35.6%)	0.02
Social isolation, *n* (%)	312 (40.7%)	108 (45.0%)	204 (38.8%)	0.10
Living alone, *n* (%)	283 (36.9%)	96 (40.0%)	187 (35.6%)	0.24
JST-IC, median (IQR)	11 (9–13)	10 (8–12)	11 (9–13)	<0.01
Number of comorbidities, median (IQR)	2 (1–2)	2 (1–2)	2 (1–2)	0.91
Polypharmacy, *n* (%)	215 (28.1%)	81 (33.8%)	134 (25.5%)	0.02
Depressive symptoms, *n* (%)	126 (16.4%)	57 (23.8%)	69 (13.1%)	<0.01
Cognitive impairment, *n* (%)	49 (6.4%)	30 (12.5%)	19 (3.6%)	<0.01

BMI, body mass index; IQR, interquartile range; JPY, Japanese Yen; JST-IC, Japan Science and Technology Agency Index of Competence; MNA®-SF, Mini Nutritional Assessment®-Short Form; oral-DDK, oral diadochokinesis; SD, standard deviation; TP, tongue pressure.

Table S2. Three logistic regression models for the association between the number of components of oral frailty and deteriorating nutritional status based on the MNA®-SF score[a]

	Outcome = having MNA®-SF score of <12 at 2-year follow-up assessment								
	Model 1 (oral frailty only)			Model 2 (Model 1 + other variables that yielded p-values < 0.05 in the crude model)			Model 3 (fully adjusted model)		
Variables[b]	ORs	95% CI	p-value	ORs	95% CI	p-value	ORs	95% CI	p-value
Number of components of oral frailty (per one increase)	1.40	(1.12–1.76)	<0.01	1.28	(1.00–1.64)	0.05	1.30	(1.01–1.68)	0.05
MNA®-SF (per one increase)				0.42	(0.28–0.63)	<0.01	0.43	(0.29–0.65)	<0.01
Age (per one increase)							1.00	(0.93–1.08)	0.97
Men (vs. women)							0.74	(0.38–1.46)	0.39
Years of schooling (per one increase)							1.02	(0.91–1.15)	0.70
Annual income < 3 million JPY							0.96	(0.45–2.06)	0.92
Current smoker							0.49	(0.15–1.56)	0.23
Daily drinker							1.48	(0.65–3.37)	0.35
Low physical activity level							1.29	(0.65–2.56)	0.46
Poor appetite				1.52	(0.83–2.80)	0.18	1.62	(0.89–2.94)	0.12
Social isolation							1.29	(0.68–2.46)	0.44
Living alone							0.62	(0.27–1.40)	0.25
JST-IC score (per one increase)				0.89	(0.80–0.99)	0.03	0.89	(0.79–1.00)	0.05

Number of comorbidities (per one increase)				1.04	(0.81–1.35)	0.74
Polypharmacy				1.07	(0.50–2.26)	0.87
Depressive symptoms	0.92	(0.40–2.14)	0.85	1.14	(0.47–2.75)	0.77
Cognitive impairment				1.99	(0.56–7.00)	0.28

CI, confidence interval; JST-IC, the Japan Science and Technology Agency Index of Competence; MNA®-SF, Mini Nutritional Assessment®-Short Form; OR, odds ratio.

[a]Applying inverse probability weighting.

[b]Except for age, sex, years of schooling, number of comorbidities, and JST-IC, ORs and CIs of being positive are presented.

Table S3. Three logistic regression models for the association between individual component of oral frailty and deteriorating nutritional status based on the MNA®-SF score[a]

	Outcome = having MNA®-SF score of <12 at 2-year follow-up assessment								
	Model 1 (component of oral frailty only)			Model 2 (Model 1 + other variables that yielded p-values < 0.05 in the crude model)			Model 3 (fully adjusted model)		
Components of oral frailty[b]	ORs	95%CI	p-value	ORs	95%CI	p-value	ORs	95%CI	p-value
1. Few remaining teeth	1.38	(0.77 to 2.48)	0.27	1.33	(0.70 to 2.52)	0.38	1.44	(0.74 to 2.78)	0.28
2. Low masticatory performance	1.50	(0.75 to 3.00)	0.25	1.39	(0.67 to 2.89)	0.38	1.50	(0.72 to 3.15)	0.28
3. Low articulatory oral motor skill	1.30	(0.61 to 2.77)	0.50	1.12	(0.52 to 2.40)	0.78	1.12	(0.51 to 2.44)	0.78
4. Low TP	1.72	(0.87 to 3.37)	0.12	1.37	(0.68 to 2.75)	0.37	1.29	(0.64 to 2.59)	0.48
5. Difficulties chewing tough foods	2.74	(1.42 to 5.26)	0.00	1.87	(0.91 to 3.85)	0.09	1.88	(0.88 to 4.04)	0.10
6. Difficulties in swallowing on tea or soup	1.44	(0.77 to 2.68)	0.25	1.29	(0.64 to 2.56)	0.48	1.32	(0.66 to 2.66)	0.43

CI, confidence interval; MNA®-SF, Mini Nutritional Assessment®-Short Form; OR, odds ratio; TP, tongue pressure

[a]Applying inverse probability weighting.

[b]ORs and CIs of being positive are presented.
